# Presence and characterization of *Escherichia coli* virulence genes isolated from diseased pigs in the central region of Argentina

**DOI:** 10.14202/vetworld.2017.939-945

**Published:** 2017-08-18

**Authors:** Fernando A. Bessone, Gabriela Bessone, Sebastián Marini, María B. Conde, Fabrisio E. Alustiza, Gustavo Zielinski

**Affiliations:** Animal Health Group, INTA - Estación Experimental Agropecuaria Marcos Juárez, Postal Code 2580, Córdoba, Argentina

**Keywords:** antibiotic resistance profile, *Escherichia coli*, pig, virulence genes

## Abstract

**Background:**

The main pathogen of neonatal and post weaning diarrhea and edema disease (ED) is *Escherichia coli* and pathotypes involved are enterotoxigenic, enteropathogenic, and shiga toxigenic (ETEC, EPEC, and STEC, respectively). Those diseases cause economic loss in pig production.

**Aim:**

The aim of this work was to evaluate the presence of strains expressing virulence markers genes and the antibiotic susceptibility profiles of *E. coli* from clinical cases of post weaning diarrhea and ED in farms in the central area of Argentina.

**Materials and Methods:**

Intensive pig farms from the central region of Argentina were sampled. Intestinal mucosa swabs from pigs with diarrhea were taken, seeded on MacConkey agar plates, biochemically typified and tested by polymerase chain reaction (PCR). Antibiograms were made by disk-diffusion method.

**Results:**

A total of 54 strains from clinical cases studied showed PCR findings: 88.88% (48/54) expressed at least one gene coding for a virulence factor. Colonization factors found were: 39.58% of strains had *F18*, 33.33% were *F4* and 31.25% adhesin involved in diffuse adherence-I; 29.17%, 25%, and 2.1% expressed *LT*, *STb*, and *STa*, respectively. 25% were *STx* and 16.67% were *eae* positive. Only 2.1% were *STx2*. The most active antibiotics against most strains were gentamicin and ceftiofur, but resistance profiles against many antibiotics were found.

**Conclusion:**

High circulation of pathogens strains of *E. coli* among pigs with diarrhea with an extended antibiotic resistance profile.

## Introduction

Porcine diarrhea is a disease that causes death and economic loss in swine production [1-5]. Enterotoxigenic, enteropathogenic, and shiga toxigenic (ETEC, EPEC, and STEC, respectively) pathotypes of *Escherichia coli* are the major pathogens responsible for neonatal diarrhea, post weaning diarrhea and edema disease (ED) in pigs [1], affecting almost all stages of production [2-4].

ETEC enterocyte adhesion is mediated by fimbriae (F) such as F4 (K88), F5 (K99), F6 (P987), F18, and F41 and is capable of producing enterotoxins which act on enterocytes. According to their thermal stability, enterotoxins are classified as heat-stable (*STa*, *STb*, and *EAST1*) and heat-labile (*LTI* and *LTII*) [5]. STEC strains produce shiga toxin (*STx1* and *STx2*) and an adhesion protein called intimin (encoded by the *eae* gene) which is responsible for microvilli attaching and effacing on enterocytes surface [6], this protein is also important in EPEC pathotype. Some strains expressing F18 and the adhesin involved in diffuse adherence (AIDA) are associated to ED and produce STx2e toxin [2,7,8]. Other strains compatible with EPEC produce intimin without expression of any other associated toxins but are capable of inducing a typical lesion of attaching and effacing microvilli on enterocytes surface [9].

Certain STEC strains, particularly enterohemorrhagic *E. coli*, may infect pigs; causing bloody diarrhea, hemorrhagic colitis, and/or hemolytic uremic syndrome in humans, which grants zoonotic importance to these strains [2]. The most important toxins produced by STEC are STx2e, EAST1, α-hemolysin, STx1, and/or STx2 [10].

Intensification of the porcine industry has led producers to carry out widespread feeding practices such as incorporation of antibiotics to feed, as a disease therapeutic strategy or as growth promoters [11]. Many antibiotics used for pig production are also important in human medicine. Dissemination of antibiotic resistance to humans might ensue from these practices [12,13]. Therefore, it is very important to know which the antibiotics resistance profiles are prevailing in the farm’s *E. coli* strains, to prevent usage of those antibiotics which are also used in human medicine but are still active against the pathogen.

Authors from different countries reported the prevalence of *E. coli* pathotypes for different categories of pigs [14-18]. However, not enough information about confirmatory diagnosis of virulent strains of *E. coli* from clinical cases is available in Argentina. Knowledge of *E. coli* pathotypes infecting the farms plus antibiotic susceptibility profiles is very important tools for pig producers to build solid therapeutic and preventive strategies.

The central aim of this work was to evaluate the presence of strains expressing virulence markers genes and the antibiotic susceptibility profiles of *E. coli* from clinical cases of post weaning diarrhea and ED occurred in farms in the central area of Argentina.

## Materials and Methods

### Ethical approval

In order to guarantee a safe, correct and carefully handling of pigs, authors proceeded according to specifications of international Ethic Guidelines (Internal Ethical Committee CICUAE Res. 533/16). However, the procedures performed do not cause discomfort to animals.

### Farms

Intensive pig production farms in the central region of Argentina (states of Córdoba, Santa Fe, Buenos Aires, Entre Ríos and San Luis, the country’s most important production area) were sampled according to the clinical cases emergency. All farms included in this study had the following characteristics: High health status, 200-500 sows, automatized feed systems, parturition control, reduced waste contact, controlled environmental temperature, and permanent veterinarian assistance.

### Bacterial isolates processing

Intestinal mucosa swabs samples were collected from clinical cases of piglets with post weaning diarrhea and/or with ED. Samples were inoculated on MacConkey agar plates (Britannia) and 5% equine blood agar plates (Britannia) to check β-hemolysis. After 24 h at 37°C Gram-staining was performed on organisms of emerging colonies to corroborate bacterial morphology. For biochemical characterization [15] hydrogen Sulfide-Indole-Motility (SIM) (Oxoid), Methyl Red-Voges Proskauer (MR-VP) (Britannia), citrate (Britannia), amino acids decarboxylation (arginine, lysine, and ornithine) (Britannia), oxidase (Britannia), catalase, three sugar iron (Oxoid) tests were carried out. All *E. coli* isolates were lyophilized until molecular characterization was made.

### Molecular characterization

A total of 54 *E. coli* isolates were tested by polymerase chain reaction (PCR). DNA was obtained using a commercial kit (Fermentas) according to manufacturer instructions for Gram-negative bacteria. These extracts were used as reaction templates. Fimbriae and toxins genes primers studied were: *STa*, *STb*, *LT*, *F4*, *F18*, *VT1* (*STx1*), *VT2* (*STx2*), *eae*, *EAST1*, *AIDA-I*, and *Rfb* (O157) [1,15], sequences are listed in Table 1. The PCR mixture was composed by 50 ng of template, 50 µM of each primer (Fagos), 100 µM deoxynucleotide triphosphates (Promega), 1.5 µM of MgCl_2_, 1× buffer (5X-Go Taq Promega), and 0.5 U of Taq Polymerase (Promega) with a final volume of 25 µL. Reactions were subjected to one initial denaturalization cycle of 94°C, followed by 30 cycles of 30 s at 94°C (denaturalization), 30 s at 58°C (primers annealing), 30 s at 72°C (extension); finally 2 min at 72°C (final extension). Reference strains of *E. coli* were used as pathotype and positive control reaction [1] (Table 1).

**Table-1:** Virulence factor genes, primer sequences.

Virulence factor	Sequence (5′-3′) – sense/antisense	Amplicon size (bp)	T° annealing (°C)
*STa*	TCC CCT CTT TTA GTC AGT CAA CTG	163	60°
	GCA CAG GCA GGA TTA CAA CAA AGT		
*STb*	GCA ATA AGG TTG AGG TGA T	368	60°
	GCC TGC AGT GAG AAA TGG AC		
*LT*	TTA CGG CGT TAC TAT CCT CTC TA	275	60°
	GGT CTC GGT CAG ATA TGT GAT TC		
*F4 (K88)*	ATC GGT GGT AGT ATC ACT GC	601	60°
	AAC CTG CGA CGT CAA CAA GA		
*F18*	GTG AAA AGA CTA GTG TTT ATT TC	510	60°
	CTT GTA AGT AAC CGC GTA AGC		
*VT1 (ST×1)*	TTA GAC TTC TCG ACT GCA AAG	530	60°
	TGT TGT ACG AAA TCC CCT CTG		
*VT2 (ST×2)*	CTA TAT CTG CGC CGG GTC TG	327	60°
	AGA CGA AGA TGG TCA AAA CG		
*eae*	CAT TAT GGA ACG GCA GAG GT	790	60°
	ATC TTC TGC GTA CTG CGT TCA		
*EAST I*	TCG GAT GCC ATC AAC ACA GT	125	55°
	GTC GCG AGT GAC GGC TTT GTA G		
*AIDA-I*	for 5×ACA GTA TCA TAT GGA GCC A	585	55°
	rev 5×TGT GCG CCA GAA CTA TTA		
*Rfb* O157	CGG ACA TCC ATG TGA TAT GG	259	55º
	TTG CTA TGT ACA GCT AAT CC		

*AIDA*=Adhesin involved in diffuse adherence

A standard electrophoresis in 2% of agarose in tris-acetate EDTA buffer stained with ethidium bromide performed at 100 V during 30 min was used to reveal the amplified products. Visualization of amplicons was made under transilluminator (UV – λ 300 nm), and a 100 pb ladder was used. Gels were photographed with a Kodak Easy Share Z7590 camera system and evaluated with Kodak Digital Science 1D software [1].

### Antimicrobial profiles

A disk-diffusion method was performed according to recommendations of the National Committee for Clinical Laboratory Standards [19]. Briefly, 12 different antibiotics were assayed: Tiamuline (TIM), enrofloxacin, ceftiofur (CTF), ampicillin, tilmicosin, erythromycin, lincomycin, espectinomicina, florfenicol, gentamicin (GEN), trimethoprim sulfamethoxazole, and tylosin. The manufacturer guidelines were used for inhibition halo interpretation. Multiple antibiotic resistances were defined as isolates showing three or more antimicrobial classes resistance [20].

### Statistical analysis

To evaluate associations between variables (genes, clinical signs, states, and season) contingency tables were used, through exact Fisher’s test. When statistical association was revealed, multivariate test “multiple correspondence analysis” was applied to know levels of variable relation. SAS version 9.2 software and InfoStat version 2015 [21] were employed.

## Results

### Bacterial isolates and molecular characterization

All isolates were biochemically compatible with *E. coli* (SIM− −/+/+; MRVP− +/−; citrate −; arginine −, lysine +; ornithine +; oxidase −; catalase +; TSI +/+). Of 54 strains from clinical cases studied, 88.88% (48/54) expressed at least one gene coding for virulence factor. PCR results showed that 87.5% (42/48) of strains expressed *EAST1*, a heat-stable toxin usually associated to enteroaggregative *E. coli*, but also associated with ETEC strains and diffuse adherence *E. coli*. Results of these findings are summarized in Table 2.

**Table-2:** Frequencies of general pathotypes distribution.

Virulence gene	Proportion	Frequency (%)*	Pathotype associated
*F18*	19/48	39.58	
*F4*	16/48	33.33	
*AIDA-I*	15/48	31.25	ETEC
*LT*	14/48	29.17	
*STb*	12/48	25	
*STa*	8/48	16.67	
*STx*	12/48	25	STEC
*ST×2*	1/48	2.1	
*eae*	8/48	16.67	EPEC

* These values are not additives, some associations between genes were found. *AIDA*=Adhesin involved in diffuse adherence, ETEC=Enterotoxigenic *Escherichia coli,* STEC=Shigatoxigenic *Escherichia coli,* EPEC=Enteropathogenic *Escherichia coli*

The most frequent combinations of virulence factors shown in Table 3. Severity of diarrhea and ED was related to *F4/EAST1 (*16.7%) and *STb/LT/F18/Stx1/East1/AIDA-I* (8.3%) combinations. On the other hand, *STb/LT/F4/EAST1 (*6.3%) was observed in strains obtained from post weaning diarrhea cases and ED. The other virulence factor combinations observed were associated with less severe disease causes.

**Table-3:** Virulence factors combination.

Genotypes combinations	Frequency (%)	Diarrhea (%)	ED (%)
*F4/East1*	8 (16.7)	4 (8.3)	4 (8.3)
*STb/LT/F18/St×1/East1/AIDA-I*	4 (8.3)	4 (8.3)	-
*STb/LT/F4/East1*	3 (6.3)	2 (4.2)	1 (2.1)
*STb/LT/F18/Eae/East1/AIDA-I*	2 (4.2)	2 (4.2)	-
*F4/Eae/East1*	1 (2.1)	-	1 (2.1)
*STa/F18/St×1/St×2/East1/AIDA-I*	1 (2.1)	1 (2.1)	-
Total	19 (39.7)	12 (27.1)	6 (12.5)

Data represent: n (%). *AIDA*=Adhesin involved in diffuse adherence, ED=Edema disease

### Antimicrobial profiles

Table 4 summarizes the results of antibiograms. According to these findings, the most suitable antibiotics for a therapeutic approach were GEN and CTF.

**Table-4:** Antibiotic profile, findings frequency.

Antibiotic	Sensitive (%)	Intermediate (%)	Resistant (%)
ENR	33.33	9.53	57.14
CEF	66.66	9.54	23.80
AMP	19.05	19.05	61.90
TIM	9.52	19.05	71.43
TIL	4.76	19.05	76.19
ERY	4.76	19.05	76.19
LIN	4.76	19.05	76.19
SPC	14.29	23.81	61.90
FFN	38.10	14.28	47.62
GEN	85.71	4.77	9.52
TMS	31.03	27.60	41.37
TYL	3.45	0	96.55

ENR=Enrofloxacin, CEF=Ceftiofur, AMP=Ampicillin, TIM=Tiamuline, TIL=Tilmicosin, ERY=Erythromycin, LIN=Lincomycin, SPC=Spectinomycin, FFN=Florfenicol, GEN=Gentamicin, TMS=Trimethoprim sulfamethoxazole, TYL=Tylosin

**Figure-1:**
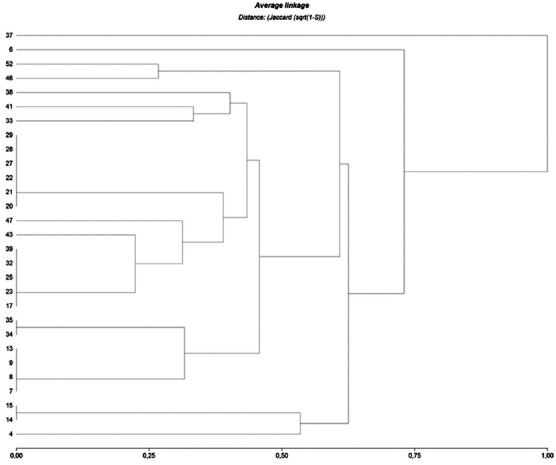
Multivariate analysis of antibiotic resistance. Dendrogram showed clusters of strains associated according to each resistance profile. Table 4 related numbers with strain.

The correspondence between isolates and antibiotics resistance was shown in Figure-1 (Table 5). Six groups of strains were observed (50% of distance), three of each included one strain distanced from the other three groups. Among these groups, one of each included two principal clusters with 23 strains; the other group was formed by two clusters with two strains each and finally one group with one strain.

**Table-5:** Association between genotype and antibiotic profile, explanation of Figure-1.

Sample ID	Genotype	Antibiotic profile		

		Sensibility	Intermediate	Resistance
4	*STb-LT-F18-eae-EASTI-AIDA-I*	CEF, AMP, FFN, GEN, TMS		TIL, TYL, ERY, LIN, SPC, OXT
6	*F4-EASTI*	ENR, CEF, AMP, FFN, GEN, OXT, TMS	TIL	TYL, ERY, LIN, SPC
7	*EASTI*	FFN, GEN		ENR, CEF, AMP, TIL, TYL, ERY, LIN, SPC, OXT, TMS
8	*F4-EASTI*	FFN, GEN		ENR, CEF, AMP, TIL, TYL, ERY, LIN, SPC, OXT, TMS
9	*F4-EASTI*	FFN, GEN		ENR, CEF, AMP, TIL, TYL, ERY, LIN, SPC, OXT, TMS
13	*F4-EASTI*	FFN, GEN		ENR, CEF, AMP, TIL, TYL, ERY, LIN, SPC, OXT, TMS
14	*STb-LT-F4-EASTI*	CEF, AMP, FFN, GEN, TMS	SPC	ENR, TIL, TYL, ERY, LIN OXT
15	*STb-LT-F4-EASTI*	CEF, AMP, FFN, GEN, TMS	SPC	ENR, TIL, TYL, ERY, LIN OXT
17	*STb-LT-F18-eae-VT1-EASTI-AIDA-I*	CEF, GEN		ENR, CEF, AMP, TIL, TYL, ERY, LIN, SPC, OXT, TMS
20	*LT-F18-EASTI-AIDA-I*	GEN	TMS	ENR, CEF, AMP, TIL, TYL, ERY, LIN, SPC, FFN, OXT
21	*VT1-EASTI*	GEN	TMS	ENR, CEF, AMP, TIL, TYL, ERY, LIN, SPC, FFN, OXT
22	*VT1-EASTI*	GEN	TMS	ENR, CEF, AMP, TIL, TYL, ERY, LIN, SPC, FFN, OXT
23	*STb-LT-F18-VT1-EASTI-AIDA-I*	CEF, GEN		ENR, CEF, AMP, TIL, TYL, ERY, LIN, SPC, OXT, TMS
25	*STb-LT-F18-VT1-EASTI-AIDA-I*	GEN	TMS	ENR, CEF, AMP, TIL, TYL, ERY, LIN, SPC, FFN, OXT
27	*EASTI*	GEN	TMS	ENR, CEF, AMP, TIL, TYL, ERY, LIN, SPC, FFN, OXT
28	*EASTI*	GEN	TMS	ENR, CEF, AMP, TIL, TYL, ERY, LIN, SPC, FFN, OXT
29	*VT1-EASTI*	GEN	TMS	ENR, CEF, AMP, TIL, TYL, ERY, LIN, SPC, FFN, OXT
32	*STb-LT-F4-ESTAI*	CEF, GEN		ENR, AMP, TIL, TYL, ERY, LIN, SPC, FFN, OXT, TMS
33	*F4-EASTI*	CEF, GEN	TMS	ENR, CEF, AMP, TIL, TYL, ERY, LIN, SPC, OXT
34	*EASTI*	CEF, GEN, TMS		ENR, CEF, AMP, TIL, TYL, ERY, LIN, SPC, OXT
35	*F4-VT1-EASTI*	SPC, FFN, GEN		ENR, CEF, AMP, TIL, TYL, ERY, LIN, OXT, TMS
37	*EASTI*	SPC, FFN, GEN		ENR, CEF, AMP, TIL, TYL, ERY, LIN, OXT, TMS
38	*STb-LTF18-VT1-EASTI-AIDA-I*	CEF, GEN, ENR, AMP, TIL, TYL, ERY, LIN, SPC, FFN, OXT, TMS		
39	*F18-EASTI*	CEF, GEN		ENR, AMP, TIL, TYL, ERY, LIN, SPC, FFN, OXT, TMS
41	*F18-EASTI-AIDA-I*	ENR, CEF, GEN, TMS		AMP, TIL, TYL, ERY, LIN, SPC, FFN, OXT
43	*F4-VT1*	CEF, GEN	FFN	ENR, AMP, TIL, TYL, ERY, LIN, SPC, OXT, TMS
46	*F4*	CEF, SPC, GEN, TMS		ENR, AMP, TIL, TYL, ERY, LIN, FFN, OXT
47	-	CEF		ENR, AMP, TIL, TYL, ERY, LIN, SPC, FFN, OXT, TMS, GEN
52	-	CEF, SPC, GEN, OXT, TMS	ENR	AMP, TIL, TYL, ERY, LIN, FFN

ENR=Enrofloxacin, CEF=Ceftiofur, AMP=Ampicillin, TIM=Tiamuline, TIL=Tilmicosin, ERY=Erythromycin, LIN=Lincomycin, SPC=Spectinomycin, FFN=Florfenicol, GEN=Gentamicin, TMS=Trimethoprim sulfamethoxazole, TYL=Tylosin, AIDA=Adhesin involved in diffuse adherence

## Discussion

This work revealed the frequency of pathotypes of *E. coli* isolated from post weaning diarrhea and ED cases in swine from the central region of Argentina, and their relation to antimicrobial susceptibility. We found combinations of virulence factors strongly associated with severity of disease [1-3,14]: *F4/EAST1 (*16.7% of strains), *STb/LT/F18/Stx1/East1/AIDA-I* (8.3%), and *STb/LT/F4/EAST1 (*6.3%); similar findings were reported by Moredo *et al*. [1,14]. However, strains expressing only one virulence factor were also found on diseased pigs, therefore those virulence factors could also be regarded as important virulence markers (e.g., *EAST1*, *STa*, *F4*, *F18*, *Stx1*, *Stx2*, and *eae*). Limited information about *E. coli* pathotypes is available in our country and it is not updated [1,14,15,22].

The results presented do not agree with those informed by Parma *et al*. [22], who reported not having found characteristic virulence genes of ETEC and STEC strains among their isolates. However, Moredo *et al*. [14] showed similar findings to ours, but in pigs without clinical signs. Genes reported by Moredo *et al*. were *STx1* (STEC) and *EAST1* (ETEC), they did not find *Rfb* O157, which was found in our work. Related to colonization factors and toxins *F18 (*40.4%), *F4 (*4.2%), *STb* (25%), and *STa* (2.1%), respectively, Moredo *et al*. [1] reported frequencies similar to ours, but we found differences regarding *LT*, which we found more frequently. These genes coding for important virulence factors have high pathogenic potential and may trigger piglet diarrhea, under adequate conditions. Therefore, the absence of disease reported by Moredo *et al*. might be related to the application of adequate environmental, hygienic, and sanitary measures [23]. Another difference was in gene coding for intimine (*eae*), not found by Moredo *et al*. [14], but detected (16.67%) in this work. In agreement with Choi *et al*. [24] *EAST1* was the most frequent virulence factor found both individually or in association with *F4*. *STb* and *LT* were genes found without any association by Choi *et al*. [24], but contrarily to that report, in our work *STb* and *LT* were found related to *EAST1*. A high proportion of *EAST1-STa* in pigs with diarrhea was detected [25], but we only found one strain with this association (Table 2). We found *F4* and *F18* in both diseases and LT in post weaning disease (PD) and one case of edema disease (ED).

The shiga toxins (*STx1*, *STx2* and its variants), intimine protein and the enterohemolysin are major virulence factors of STEC [26]. Although no O157 gene was found, we obtained a prevalence of 25% for *STx1*, 16.67% for *eae* (gene encoding intimine protein), and 2.1% for *STx2*. This fact suggests that pigs with post weaning diarrhea or ED could be reservoirs of pathogenic strains of *E. coli* [9]. Pigs carrying hemolytic uremic syndrome (HUS)strains could potentially generate an outbreak of that disease not only by possible environmental contamination but also because of participation of pork in the food chain. Fortunately, such strains were not found in this study.

Bacteriological studies of clinical cases of diarrhea in swine production should be standard practice. However, in developing countries, diarrhea treatment is frequently intuitive or empiric with concomitant multiresistance dissemination [20]. Our results showed important antibiotic resistant profiles among isolates from sick animals such as Okello *et al*. [12], Luppi *et al*. [13], and Mathew *et al*. [11]. GEN and CTF elicited low amounts of resistant strains in accordance with Moredo *et al*. [1], who found a few antibiotics inactive for ETEC strains from healthy pigs. Dendrograms are useful tools to evaluate distinct drugs or groups of antibiotic resistance in a same pathotype of bacteria.

## Conclusion

In conclusion, we provide information about an important circulation of strains and pathotypes of *E. coli* among pigs with diarrhea. The strains studied showed high resistance profiles, these results could be useful for swine industry.

## Authors’ Contributions

Conceived and designs the experiments: FB, FA, and GZ. Perform the experimental procedures: FB, GB, SM, and FA. Analyzed the data: FB, FA, and MC. Wrote the paper: FB, FA, and GZ. All authors read and approved the final manuscript
